# Paper-Based Microfluidic Device for Extracellular Lactate Detection

**DOI:** 10.3390/bios14090442

**Published:** 2024-09-14

**Authors:** Yan Nan, Peng Zuo, Bangce Ye

**Affiliations:** Lab of Biosystem and Microanalysis, State Key Laboratory of Bioreactor Engineering, East China University of Science & Technology, Shanghai 200237, China

**Keywords:** paper-based microfluidic, lactate detection, colorimetric analysis, tumor cell, drug screening

## Abstract

Lactate is a critical regulatory factor secreted by tumors, influencing tumor development, metastasis, and clinical prognosis. Precise analysis of tumor-cell-secreted lactate is pivotal for early cancer diagnosis. This study describes a paper-based microfluidic chip to enable the detection of lactate levels secreted externally by living cells. Under optimized conditions, the lactate biosensor can complete the assay in less than 30 min. In addition, the platform can be used to distinguish lactate secretion levels in different cell lines and can be applied to the screening of antitumor drugs. Through enzymatic chemical conversion, this platform generates fluorescent signals, enabling qualitative assessment under a handheld UV lamp and quantitative analysis via grayscale intensity measurements using ImageJ (Ver. 1.50i) software. The paper-based platform presented in this study is rapid and highly sensitive and does not necessitate other costly and intricate instruments, thus making it applicable in resource-constrained areas and serving as a valuable tool for investigating cell lactate secretion and screening various anti-cancer drugs.

## 1. Introduction

Lactate, a pivotal metabolic byproduct of anaerobic glycolysis, has garnered significant importance in clinical medicine, exercise physiology, and food science [1]. Traditionally perceived as a metabolic waste associated with muscle fatigue [2], recent research has unveiled lactate’s critical roles in both physiological and pathological cellular states. Approximately 1500 mM of lactate is produced daily from various tissues, including skeletal muscle, heart, and brain [3]. It serves not only as an energy substrate, supplying energy [4], but also participates in energy transfer processes within and between tissues. Converted into pyruvate, lactate contributes to oxidative phosphorylation and ATP generation through the tricarboxylic acid cycle [5]. Beyond its role as an energy source, lactate functions as a signaling molecule, influencing processes such as tumor cell proliferation, immune evasion, and neuronal energy metabolism [6].

Disruptions in lactate production and consumption can lead to various diseases, highlighting the critical importance of measuring blood lactate concentrations for diagnosis and treatment [7]. In sports medicine, lactate serves as a signaling molecule that positively regulates metabolic processes during physical activity. Blood lactate concentration sensitively reflects changes in exercise intensity and duration, making it a vital metric for evaluating an athlete’s training level [8]. Furthermore, during physical exertion, lactate serves as an alternate energy substrate for the brain, conserves glucose and stimulates the hypothalamus to regulate energy intake and neuronal activity, thereby playing a pivotal role in enhancing brain metabolism and overall function [9]. In food analysis, monitoring lactate levels is crucial for evaluating the quality, freshness, and preservation stability of various food products [10], including fruits, meats, alcoholic beverages [11], and specific fermented dairy items [12]. Given lactate’s association with inflammatory states, cancer, and other health issues, its precise control and detection within the food sector are paramount.

Tumor cells undergo aerobic glycolysis and produce lactate even in the presence of sufficient oxygen, a phenomenon known as the “Warburg effect.” [13]. Lactate accumulation within the tumor microenvironment significantly impacts tumor progression by fostering cell invasion and angiogenesis [14]. While lactate concentrations are tightly regulated at approximately 1.5–3 mM under normal physiological conditions [15], they can escalate to 30 mM within tumor microenvironments, potentially influencing cellular functions [16]. Tumor cells rapidly respond to metabolic signals in the environment and increase energy metabolism by regulating mitochondria-associated pathways and the TCA cycle, thereby promoting tumor growth and metastasis [17]. Consequently, lactate serves not only as a crucial player in energy metabolism but also as a significant regulator of tumor development [18]. Accurate and sensitive analysis of lactate secretion by tumor cells holds promise for early cancer diagnosis.

Due to the crucial regulatory role of lactate in tumor occurrence and development, accurate and reliable analysis and detection are essential. The main techniques for lactate detection include chromatographic analysis, fluorescence detection, luminescence methods, and electrochemical methods. Common chromatographic analysis methods used for lactate detection include high-performance liquid chromatography (HPLC), mass spectrometry (MS), gas chromatography (GC), and spectroscopic methods. These techniques quantitatively analyze lactate by separating and detecting the concentration of lactate present in the sample. Omar Kadi et al. proposed an LC-MS/MS method for concurrent detection and quantification of crucial metabolites in various cancer types like prostate cancer. This approach involves extracting and qualitatively and quantitatively analyzing glutamine, citrate, isocitrate, malate, succinate, fumarate, and lactate from body fluids, tissues, and human-derived cultured cell lines [19]. Fluorescence methods are analytical techniques based on the interaction between the analyte and a fluorescent dye or label, producing a fluorescent signal. A study [20] designed a glass capillary platform for fluorescence detection of lactate. The inner wall of the glass capillary is patterned with lipids and bovine serum albumin (BSA) modifications, utilizing electrostatic interactions to immobilize LDH on different regions of the lipid layers. It was found that the fluorescence intensity at the enzyme sites increases with the increasing L-lactate concentration, exhibiting a positive correlation. The established method has a detection limit of 4.9 μM for L-lactate. Electrochemical methods utilize the detection system to record and analyze changes in electrical signals, which are then converted into the concentration or activity of the target molecule. M. Briones et al. [21] designed an electrochemical biosensing platform by modifying a gold electrode with undoped diamond nanoparticles and employing LOx as a model enzyme for lactate detection. This platform exhibits a linear concentration range of 0.05–0.7 mM, a sensitivity of 4.0 μA/mM, and a detection limit of 15 μM. Although accurate lactate detection can be provided based on the techniques described above, these techniques require a significant investment of time, complex preparation processes, and expensive equipment, which limits their widespread use, especially in point-of-care (POC) testing in less developed regions. Biosensors currently outperform traditional detection methods [21], offering alternative solutions to circumvent the constraints of conventional techniques. Paper-based microfluidics is an emerging technology in the field of microfluidics, primarily utilizing porous materials like paper as the substrate for microfluidic chips, with capillary action driving the flow and diffusion of liquids within the paper. By designing specific hydrophobic/hydrophilic regions on the paper, the flow path and diffusion areas of the liquid can be controlled. Paper-based microfluidic chips can be used for various biochemical analyses, such as enzymatic reactions, immunoassays, and nucleic acid detection. By pre-immobilizing reagents in specific regions, the reaction can occur as the test liquid flows through, and the results can be read visually or using instruments. Paper-based chips can be stored for long periods of time due to their stabilizing properties. Due to its low cost, portability, and ease of operation, paper-based microfluidics has garnered significant attention in the field of lactate detection. Combining paper-based microfluidics with the catalytic reaction of lactate oxidase can enable the detection and analysis of lactate.

Based on the limitations of the above detection methods, this study combined the advantages of paper-based microfluidics to develop a paper-based microfluidic platform for lactate detection integrating the paper-based microarray previously developed by our team [22]. Based on the chip’s ability to culture cells in three dimensions, the detection of exocrine lactate from living cells was realized. Although lactate lacks color characteristics or fluorescence absorption, its reaction with LDH and NAD^+^ produces NADH through dehydrogenation, which can be detected through fluorescence [23]. This method allows lactate detection within 30 min, providing a new avenue for early tumor diagnosis. Furthermore, we investigated the differences in lactate secretion levels between tumor and normal cells, which is crucial for early tumor detection. Additionally, this portable microdevice enables imaging and colorimetric analysis of drug screening results, showing potential applications in effective drug screening and biomedical research.

## 2. Materials and Methods

Lactate was obtained from Shanghai Titan Scientific Co., Ltd. (Shanghai, China), LDH was obtained from Ying Xin Laboratory Equipment Co., Ltd. (Shanghai, China), and NAD^+^ was obtained from Yi sheng Chemical Technology Co., Ltd. (Shanghai, China). The commercial lactate detection kit was provided by Elabscience Biotechnology Co., Ltd. (Wuhan, China), and paclitaxel was purchased from Dibai Biotechnology Co., Ltd. (Shanghai, China). Doxorubicin hydrochloride was obtained from Bide Pharmatech Ltd., (Shanghai, China). Collagen was purchased from Sigma Aldrich, Inc. (Saint Louis, MO, USA). Dulbecco’s modified eagle medium (DMEM), fetal bovine serum (FBS), and trypsin-EDTA were purchased from Thermos Fisher Scientific Inc. (Waltham, MA, USA). CCK-8 was obtained from TransGen Biotech Co., Ltd. (Shanghai, China). PMHS was provided by Darui Chemicals Co., Ltd. (Shanghai, China). All other chemicals were of analytical grade and used as received.

The filter paper of Grade 1 (particle retention in liquid: 11 µM; thickness: 180 µM) and Grade 4 (particle retention in liquid: 20 to 25 µM; thickness: 200 µM) were purchased from Whatman (London, UK). An inkjet printer (TS3150) was purchased from Cannon Co., Ltd. (Shanghai, China). A 24-well microplate (Corning Inc., Corning, NY, USA) served as the medium container in a paper-based microfluidic platform. Chromogenic results were imaged using a smartphone camera.

Human breast adenocarcinoma cell line (MCF-7), hepatocellular carcinoma cell line (HepG2), Metastatic Breast-231 cell line (MB-231), human embryonic kidney 293T cell line (HEK293T), HeLa cell line, and normal human fetal liver cells (L-02) were obtained from the Cell Bank of Type Culture Collection of the Chinese Academy of Sciences (Shanghai, China). Cells were cultured using a standard cell culture technique for 2 or 3 days, followed by digestion with 0.05% trypsin for 2 min and centrifugation at 8000× *g* for 2 min. The cell pellet was resuspended in the culture medium and saved for further use.

### 2.1. Production and Optimization of the Paper-Based Microfluidic Platform

This study utilized an inkjet printer to prepare paper-based chips for lactate detection. A hydrophobic ink was prepared by mixing polydimethylsiloxane (PMHS) and n-butanol in a 2:1 ratio and filtering through a 0.22 μm filter. The filtered ink was then loaded into a clean ink cartridge. The pattern design was created using Adobe Illustrator (Adobe Inc., San Jose, CA, USA) and subsequently printed onto the paper using an inkjet printer, with the printed area serving as the hydrophobic section. Following printing, the paper was subjected to a 65 °C oven to facilitate crosslinking of PMHS on the paper surface. The paper-based cell culture microfluidic platform was developed based on prior designs by our group, comprising two stacked layers of filter paper. A hydrophobic pattern was printed on the paper to delineate a closed hydrophilic zone conducive for cell growth. The two paper sheets were then bonded together using tape, aligning identical circular patterns. Subsequently, the hydrophilic channel was folded at a 90° angle to facilitate medium delivery. Finally, the paper device underwent overnight UV disinfection in preparation for subsequent experimental use.

To minimize the interference caused by the paper-based substrate’s background color in the fluorescence signal detection and facilitate direct visual detection of low-concentration target product fluorescence signals, an optimization of the paper-based background color was performed. For this purpose, red, yellow, purple, and black paper sheets were used for optimization experiments, with each sheet undergoing hydrophobic treatment. The experimental process involved applying NADH standard substance (10 mM, 20 mM, 30 mM, 40 mM, and 50 mM) onto the paper sheets, followed by detecting the fluorescence signals emitted by the experimental groups under a portable ultraviolet lamp. Fluorescence signals were photographed, and the images were processed using ImageJ to convert them into grayscale values. By analyzing the linear relationship between fluorescence signals on different color paper sheets, the optimal background color was determined. To ensure the reliability of the research results, consistent image acquisition conditions were maintained throughout all experiments, thereby improving the accuracy of the detection method.

### 2.2. Optimization of the Paper-Based Lactate Detection Platform

The feasibility of a fluorescence assay method for lactate detection was validated through fluorescence intensity measurements on a microplate reader. The following experimental groups were analyzed: (1) 100 μL Tris-HCl buffer (pH = 7); (2) 100 μL LDH; (3) 100 μL NAD^+^; (4) 50 μL LA + 50 μL LDH; (5) 50 μL LA + 50 μL NAD^+^; (6) 50 μL LDH + 50 μL NAD^+^; and (7) the positive control sample containing 50 μL LA + 25 μL LDH + 25 μL NAD^+^. All solutions were prepared in Tris-HCl buffer and added to the detection zone. The samples were then incubated at 37 °C for 30 min, exposed to a UV lamp, and the fluorescence of the reaction products was captured by photography. The gray-scale values of the images were analyzed using ImageJ (Ver. 1.50i) software.

For the paper-based detection platform, we optimized the system based on four conditions, including the concentrations of LDH and NAD^+^, reaction pH, and reaction time. Initially, the lactate concentration was set at 100 mM, NAD^+^ concentration was set at 500 mM, and the pH of Tris-HCl buffer was set at 7. An LDH concentration gradient ranging from 1000 to 6000 U L^−1^ was established. The reaction mixture was added to the reaction zone of the paper chip and incubated at 37 °C for 30 min to facilitate the reaction. Subsequent observation and photography were conducted in a light-proof setting using a portable UV lamp for further analysis. Furthermore, optimization of NAD^+^ concentration (450–500 mM), buffer pH (7–10), and reaction time (5–30 min) was performed. During optimization, all parameters except the one under investigation were kept constant to ensure result reliability. A consistent photographic environment was maintained throughout all experiments to improve assay accuracy.

### 2.3. Establishment of the Paper-Based Lactate Detection Platform

To establish the relationship between fluorescence intensity and lactate concentration, quantitative analysis was performed using lactate standards at concentrations of 100 mM, 200 mM, 300 mM, 400 mM, 500 mM, and 600 mM. By capturing images and analyzing the fluorescence signals with specialized software, standard curves were generated, enabling precise quantification of lactate concentration based on fluorescence intensity. Furthermore, a commercial lactate assay kit was used to be compared with the paper-based lactate detection method. Lactate samples at various concentrations (100, 200, 300, 400, 500, and 600 mM) were tested to generate a calibration curve, following the manufacturer’s instructions. The kit uses NAD^+^ as a hydrogen acceptor, with LDH facilitating the conversion of lactate to pyruvate, thereby transforming NAD^+^ into NADH. In this process, N-methyl phenazine methyl sulfate transfers hydrogen to reduce nitro tetrazolium blue chloride to a purple colorant, which can be quantified by measuring the optical density.

### 2.4. Detection of Lactate Secreted by Tumor Cells Using the Paper-Based Platform

In this study, a 3D paper-based culture platform was developed for cell cultivation and the detection of cell-secreted lactate. Six different cell types (MCF-7, HepG2, MB231, HEK293T, HeLa, and L-02) were seeded onto the culture area and incubated at 37 °C with 5% CO_2_. The reaction system, which included 5000 U L^−1^ of LDH and 500 mM NAD^+^, was added to the culture zone after incubation. The mixture was then incubated at 37 °C, and images were collected and analyzed under light avoidance conditions. Cell viability in the culture area was evaluated using the CCK-8 assay, with color intensity representing cell viability and cytotoxicity.

### 2.5. Applications for the Paper-Based Lactate Detection Platform

Two anticancer drugs were prepared in DMEM following dissolution in DMSO, with DMSO concentrations ranging from 0.1% and 0.8%, a range deemed safe due to its toxicity being below 1%. The drug-containing medium was applied to the culture zone, saturating the paper-based chip, followed by the addition of MCF-7 cell suspension to each zone. The paper device was incubated at 37 °C for 24 h. After incubation, the lactate detection system was added to the incubated zones and further incubated at 37 °C for a predetermined time to facilitate reaction. Similarly, the CCK-8 re-agent was applied to the paper device to evaluate cell viability, providing a comprehensive assessment of the effects of the anticancer drugs on the cells.

Results were analyzed by one way analysis of variance (ANOVA) using GraphPad Prism 7 (GraphPad Software Inc., CA, USA). Data are presented as the mean ± standard deviation (SD) more than 3 independent experiments. Statistically significant was determined based on a *p*-value of less than 0.05 (* *p* < 0.05, ** *p* < 0.01, *** *p* < 0.001, and **** *p* < 0.0001; n = 3). It mathematically determines the difference between two experimental data set, and the baseline is not due to random chance.

## 3. Results

### 3.1. Subsection

#### 3.1.1. Principle of Lactate Detection on the Paper-Based Microfluidic Platform

**Figure 1:**
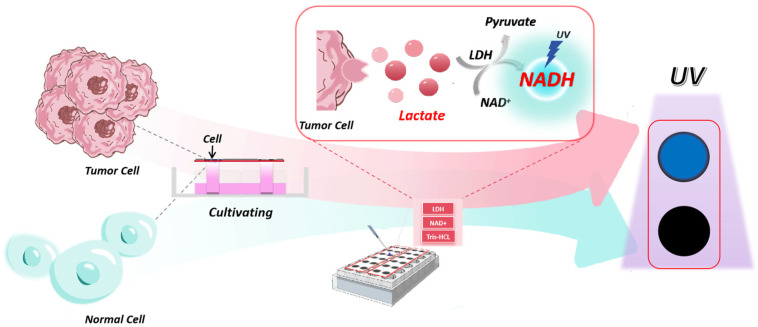
Principle of the paper-based microfluidic platform. This paper-based microfluidic device combines the function of both analysis and detection.

Figure 1 illustrates the schematic diagram of the entire detection process. The design schematic of the chip is shown in Supplementary Materials (Figure S1). Building upon previous research in our laboratory, the reaction solution containing LDH and NAD^+^ was added to the reaction zone on the paper-based platform, which was used for 3D cell culture. Following co-incubation and redox reactions, the plate was placed into a customized light-shielding device. The fluorescent product was then illuminated with a handheld UV lamp, and the information was captured using a smartphone camera. Finally, ImageJ software was utilized for data quantification. Since the detection on this platform occurred at the endpoint of the lactic acid detection reaction and image acquisition could be completed within 1 min, the ultraviolet irradiation from the portable UV lamp had minimal impact on cell viability.

Different concentrations of NADH standard solutions (10, 20, 30, 40, and 50 mM) were placed on the paper-based chips, and the captured fluorescence images were analyzed by using ImageJ through grayscale processing (Figure S2). The blue channel exhibited the highest sensitivity when collecting and analyzing image information (Figure S2B). Therefore, we chose to utilize the blue channel for signal collection and analysis. Figure S3 illustrates the correlation coefficients for each background color: R^2^ = 0.9871 for red, R^2^ = 0.9134 for yellow, R^2^ = 0.9774 for purple, and R^2^ = 0.9943 for black. As shown in Figure S3D, samples with different concentration gradients displayed distinct color changes only on black paper, minimizing interference from the paper color. Consequently, black was chosen as the optimal background color for the paper-based platform.

#### 3.1.2. Validation of the Feasibility of Lactate Detection Using the Paper-Based Platform

**Figure 2:**
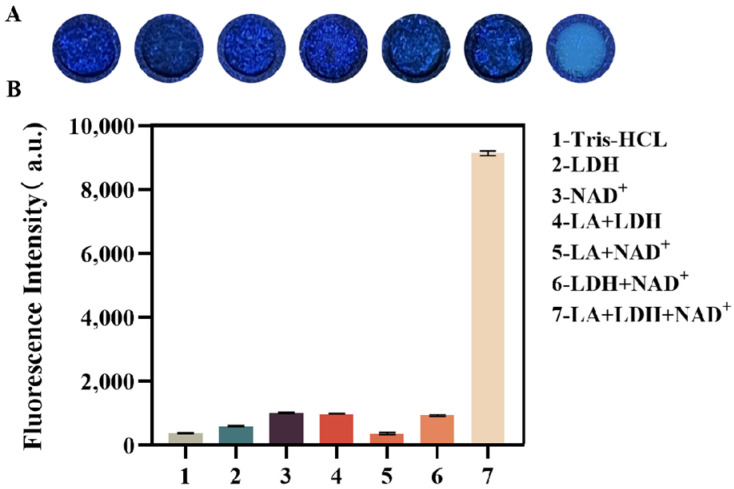
Validation of the feasibility of lactate detection using the paper-based platform. (**A**) The fluorescence images of the products from the reactions of different reagent combinations after 30 min (shot under a handheld UV lamp). (**B**) Fluorescence measurements of different combinations of the reagents with a microplate reader. Combination 1: the blank control; 2: LDH; 3: NAD^+^; 4: LA+LDH; 5: LA+NAD^+^; 6: LDH+NAD^+^; 7: LA+LDH+NAD^+^. All solutions were in Tris-HCl buffer.

As shown in Figure 2, lactate can only be converted to NADH and produce a visible fluorescent signal when both LDH and NAD^+^ are present. Figure 2 displays seven sets of fluorescent signals evaluated using a microplate reader on the chip. The fluorescence values measured by the plate reader confirmed that the fluorescence signals generated by each group were consistent with the color intensity observed by the naked eye, indicating that the paper-based platform is suitable for lactate detection. Notably, the fluorescence intensity of the experimental group was approximately 10 times higher than that of the control group samples.

**Figure 3:**
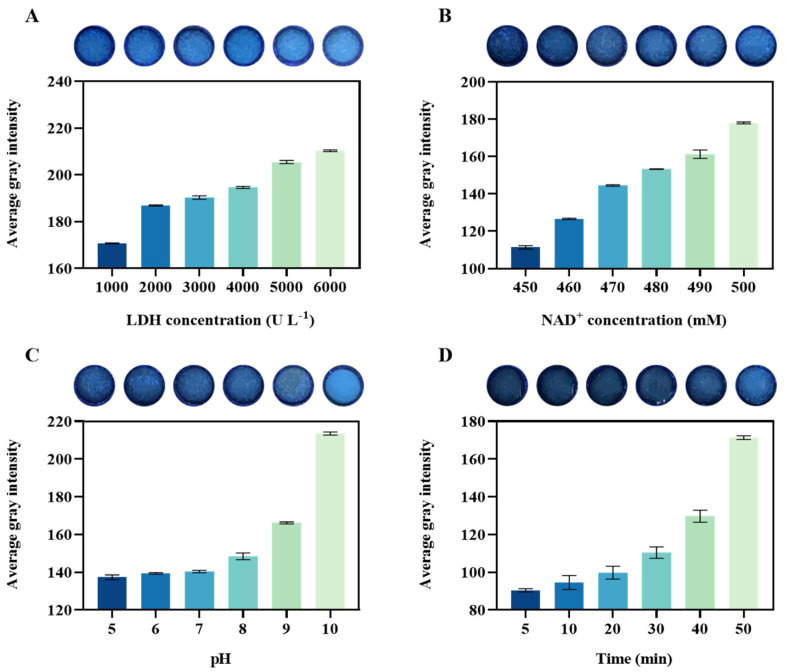
Optimization of the fluorescence assay for the detection of lactate. (**A**) Optimization of LDH concentration. (**B**) Optimization of NAD^+^ concentration. (**C**) Optimization of buffer pH value. (**D**) Optimization of reaction time for the detection system. Each group includes fluorescence images taken under various conditions, alongside corresponding histograms showing grayscale signals. All the evaluations were carried out in triplicate (n = 3).

Next, the correlation between LDH concentration and catalytic reaction was preliminarily explored. As shown in Figure 3A, when the NAD+ concentration was fixed, the fluorescence intensity gradually increased with LDH concentration ranging from 1000 U L^−1^ to 5000 U L^−1^. However, there was a minimal change in fluorescence signal at LDH concentrations between 5000 U L^−1^ and 6000 U L^−1^, indicating saturation at these levels. Thus, an LDH concentration of 5000 U L^−1^ was sufficient for the detection purposes. Meanwhile, the graph in Figure S4A shows the fluorescence quantification of the experimental group using a microplate reader. The results demonstrate a linear correlation between LDH concentration and fluorescence signal intensity. Similarly, Figure 3B shows the optimization of NAD^+^ concentration. When the LDH concentration was fixed at 5000 U L^−1^, adjusting the NAD^+^ concentration led to changes in fluorescence in the reaction, consistent with the above results. Experimental data indicate that the saturation concentration of NAD^+^ was 500 mM. Figure S4B quantitatively evaluates the fluorescence of the experimental groups using a microplate reader. The results are consistent with the linear relationship between experimental concentration and fluorescence intensity. Therefore, the optimal NAD^+^ concentration for significant fluorescence response was 500 mM. Consequently, we selected 5000 U L^−1^ and 500 mM as the reaction concentrations for LDH and NAD^+^, respectively. The production of NADH in the catalytic reaction led to changes in H^+^ concentration, indicating that adjusting the pH with Tris-HCl affects the fluorescence results of the catalytic reaction. Figure 3C shows that when the system’s pH value started to shift from pH 7 to alkaline conditions, the fluorescence intensity rapidly increased. This suggests that the detection system requires a stable pH buffer solution, so a buffer with a pH of 7 was chosen for the experiments. Furthermore, we determined the optimal reaction time for the detection method by analyzing data at different reaction times (0–50 min), as shown in Figure 3D. As can be seen from the figure, a faint fluorescence can be seen at the fifth minute, indicating that the paper-based platform can rapidly detect lactic acid. Considering the best visible effect, we set the reaction time for paper-based lactate detection to 30 min.

#### 3.1.3. Sensitivity of the Assay

**Figure 4:**
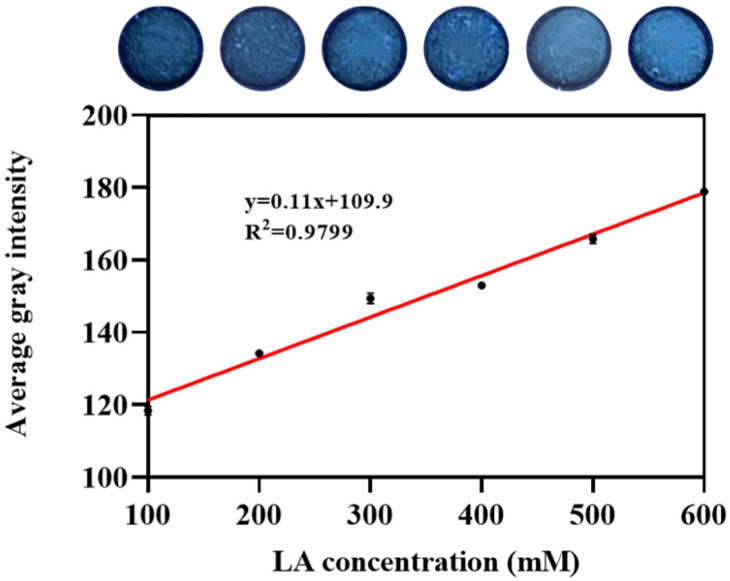
Sensitivity of lactate detection based on the paper-based platform. Fluorescence photographs of lactate detection at different concentrations (i.e., 100 mM, 200 mM, 300 mM, 400 mM, 500 mM, and 600 mM) on the paper-based platform are shown. The calibration curve by plotting the average grayscale values against the different lactate concentration is presented. Error bars: standard deviation from different experiments (n = 3).

Varying lactate concentrations (100, 200, 300, 400, 500, and 600 mM) were served as reaction templates. Plotting the gray level against lactate concentration produced a linear curve with an R^2^ = 0.9799 (Figure 4), indicating a strong linear relationship across each experimental group and the detection limit of the assay was 5.6699 mmol L^−1^ (S/N ¼ 3). Microplate reader analysis (Figure S5A) revealed a direct proportionality between fluorescence intensity and lactate concentration. Compared to a commercial lactate detection kit (R^2^ = 0.9746; Figure S5B), our paper-based microfluidic platform exhibited a similar standard curve. This approach resulted in a standard curve with a detection limit of 5.6834 mmol L^−1^ (S/N ¼ 3), as depicted in Figure S5B. This comparison demonstrates that our paper-based microfluidic analysis achieves a comparable detection limit to that of commercial kits.

#### 3.1.4. Applications for the Paper-Based Lactate Detection Platform

**Figure 5:**
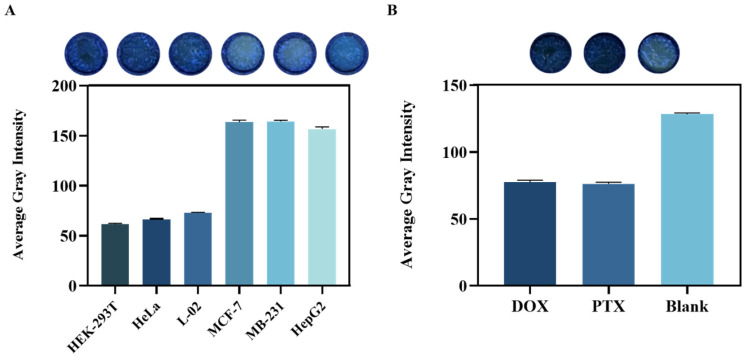
Applications for the paper-based lactate detection platform. (**A**) Cultivation of different types of cells on the paper-based platform and lactate detection: fluorescence images were taken after the reaction of the lactate detection system 24 h after cell culture; (**B**) Lactate secretion of MCF-7 cells under different drug treatments (doxorubicin and paclitaxel at a concentration of 10 µg mL^−1^, as well as without treatment) were examined on the paper-based platform. Fluorescence images (arranged left to right: doxorubicin, paclitaxel, and no treatment) were used to derive grayscale values for the histograms. Error bar: standard deviation from different experiments (n = 3).

We integrated a lactate detection system into our previously established 3D cell culture paper-based microfluidic platform to investigate lactate secretion variations across cell types and evaluate the platform’s specificity. Feasibility of culturing MCF-7 cells on the chip was demonstrated (Figure S6A), with CCK-8 assay data showing increased cell viability with higher seeding densities. Consequently, 16,000 cells were seeded for subsequent experiments. Cell viability analysis after seeding 16,000 cells for varying durations (Figure S6B) led to a 24 h culture period being chosen to ensure sufficient cell numbers for lactate detection. Figure 5A demonstrates the assessment of lactate secretion across six cell types (normal: HEK 293T, HeLa, and L-02; tumor: MCF-7, MB231, and HepG2) using the paper-based platform, confirming its specificity. Distinct fluorescence intensities and grayscale values were observed for tumor cells, indicating varying lactate secretion levels and the platform’s ability to sustain and detect continuous lactate secretion. In contrast, negligible fluorescence signals were observed for normal cells under identical culture conditions, suggesting significantly lower lactate secretion. Quantification based on the established standard curve revealed a lactate secretion rate of approximately 330 mM for tumor cells.

Paclitaxel and doxorubicin were employed as anticancer agents against MCF-7 cells, and the paper-based microfluidic platform facilitated quantitative analysis of lactate secretion from drug-treated cells, with color intensity directly reflecting lactate production. Figure 5B shows a notable decrease in fluorescence intensity and grayscale values in the drug-treated groups compared to in the untreated control, indicating significant reduction in lactate secretion from MCF-7 cells post-treatment (*p* < 0.05). These findings highlight the drugs’ ability to markedly diminish lactate production by impeding tumor cell proliferation, demonstrating the device’s sensitivity to paclitaxel and doxorubicin, corroborated by CCK8 assay data (Figure S7) showing decreased cell viability in the drug-treated groups, further underscoring the platform’s utility in drug screening.

## 4. Conclusions

In this study, we introduced a paper-based microfluidic platform for lactate detection, which was further integrated with a wick-type paper microfluidic cell culture device. This combination enabled the detection of lactate secretion and screening of drugs targeting tumor cells. Leveraging an enzyme-assisted chemical reaction for lactate within the paper-based microfluidic platform, this approach offers biocompatibility, affordability, and operational simplicity. Others have also detected lactate secreted inside and outside of cells. Marcel Braendlein [24] used organic transistor circuits to detect lactate in tumor cell cultures, and Yuanyu Zhang [25] et al. used a multi-enzyme system in an amorphous metal−organic framework to detect lactate inside cells. Although these methods can also detect lactate, they are more expensive compared to the paper-based platform we have built and require trained specialists to operate the experiments. The method employs image acquisition for fluorescence signal collection, providing an accessible, cost-effective, efficient, and reliable technique for lactate measurement. The sensitivity and specificity of this microfluidic paper-based lactate detection platform was experimentally validated. Moreover, when combined with a smartphone-based colorimetric analysis system, the paper-based microfluidic device emerges as a powerful tool for examining lactate secretion from cells and screening various anticancer drugs.

## Data Availability

No new data were created.

## Supplementary Materials

The following supporting information can be downloaded at: https://www.mdpi.com/article/10.3390/bios14090442/s1, Figure S1. Detail of the paper-based chip design diagram. (A) The size dimension of the 12-circle paper chip. (B) The 3D model of 12-circle paper chip. Figure S2. Optimization of Grayscale Analysis Channels. (A) Effect images of processing fluorescent products under four different grayscale channels. (B) The relation curves between grayscale values and NADH concentration under different grayscale analysis channels. Figure S3. Color optimization of paper chip. The linear relationship and correlation coefficient between the NADH concentration and the corresponding gray value of fluorescence signal in the experimental group using red paper chip (A), yellow paper chip (B), purple paper chip (C) and black paper chip (D). All the evaluations were carried out in triplicate (n = 3). ∗ represents a significant difference among the groups with p < 0.05. Figure S4. Conditions and fluorescence value curves of each experimental group in the microplate reader. (A) Optimization of LDH concentration. (B) Optimization of LDH concentration. NAD+ concentration. (C) Optimization of buffer pH value. (D) Optimization of reaction time. Error bars: standard deviation from different experiments (n = 3). Figure S5. Calibration of the standard curve of fluorescence and lactate concentration using a microplate reader. Error bars: standard deviation from different experiments (n = 3). (B) Calibration of the standard curve of absorbance and lactate concentration in microplate wells using a commercial kit. Error bars: standard deviation from different experiments (n = 3). Figure S6. Feasibility verification of cell culture on paper-based platform (A) Calibration curve of different cell seeding densities and average gray intensity. (B) Calibration curve of cell culture days and average gray intensity. The average gray intensity was measured by the CCK-8 assay, and the image information was acquired by a smartphone. The results show that cell viability increases with increasing cell density. Considering the adhesion area of cells on paper, a seeding density of 16,000 cells/mL was chosen. As the culture time increased, the cell density also increased. To ensure sufficient cell density, cells were cultured for 24 h after seeding 16,000 cells for subsequent experiments. Additionally, it can be observed that our paper-based platform can be stored for at least 5 days for experimentation. All the evaluations were carried out in triplicate (n = 3). Figure S7. The cytotoxic effect of MCF-7 cells under the treatment of doxorubicin and paclitaxel: Cell viability detection after treatment with different drugs on a paper-based platform (images from left to right, doxorubicin, paclitaxel, concentration at 10 µg mL^−1^); Histogram showing grayscale intensity, collected by Image J from the images. All evaluations were repeated three times (n = 3).
