# Supplementary material for: Paper-Based Microfluidic Device for Extracellular Lactate Detection
Source: Biosensors (Basel). 2024 Sep 14;14(9):442. doi: 10.3390/bios14090442 (PMC11430598; doi:10.3390/bios14090442)
Supplement: Supplementary file 1 [file biosensors-14-00442-s001.zip › biosensors-3066491-supplementary.pdf]

biosensors

Supporting information

Paper-Based Microfluidic Device for Extracellular Lactate Detection

Yan Nan, Peng Zuo * and Bang-Ce Ye *

Lab of Biosystem and Microanalysis, State Key Laboratory of Bioreactor Engineering, East China University of Science & Technology, Shanghai 200237, China
* Correspondence: pzuo@ecust.edu.cn (P.Z.); bcye@ecust.edu.cn (B.-C.Y.)

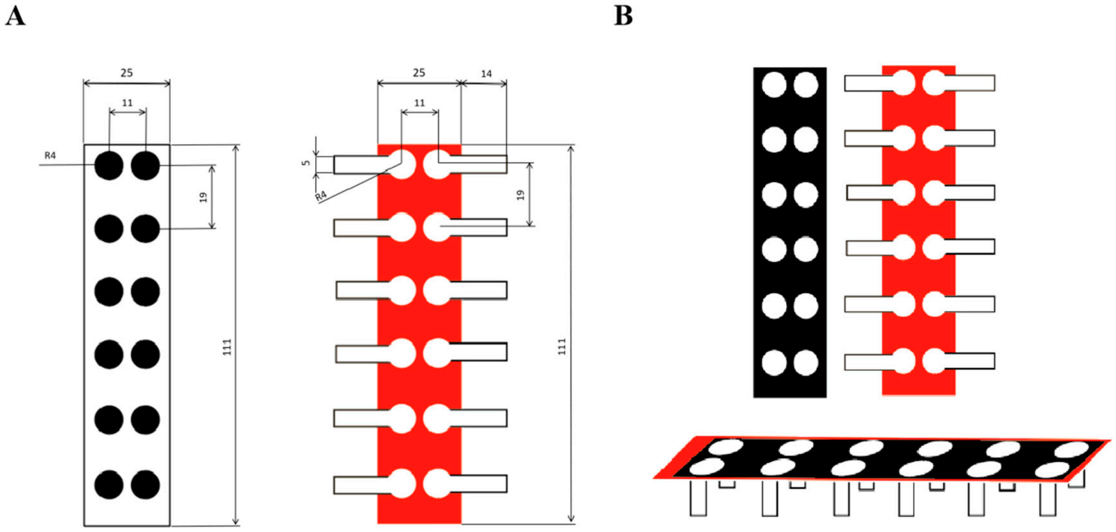

Figure S1. Detail of the paper-based chip design diagram. (**A**) The size dimension of the 12-circle paper chip. (**B**) The 3D model of 12-circle paper chip.

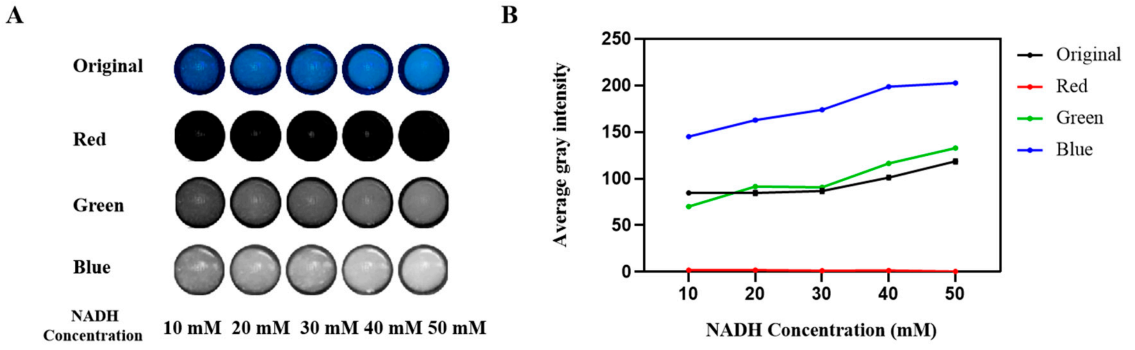

Figure S2. Optimization of Grayscale Analysis Channels. (**A**) Effect images of processing fluorescent products under four different grayscale channels. (**B**) The relation curves between grayscale values and NADH concentration under different grayscale analysis channels.

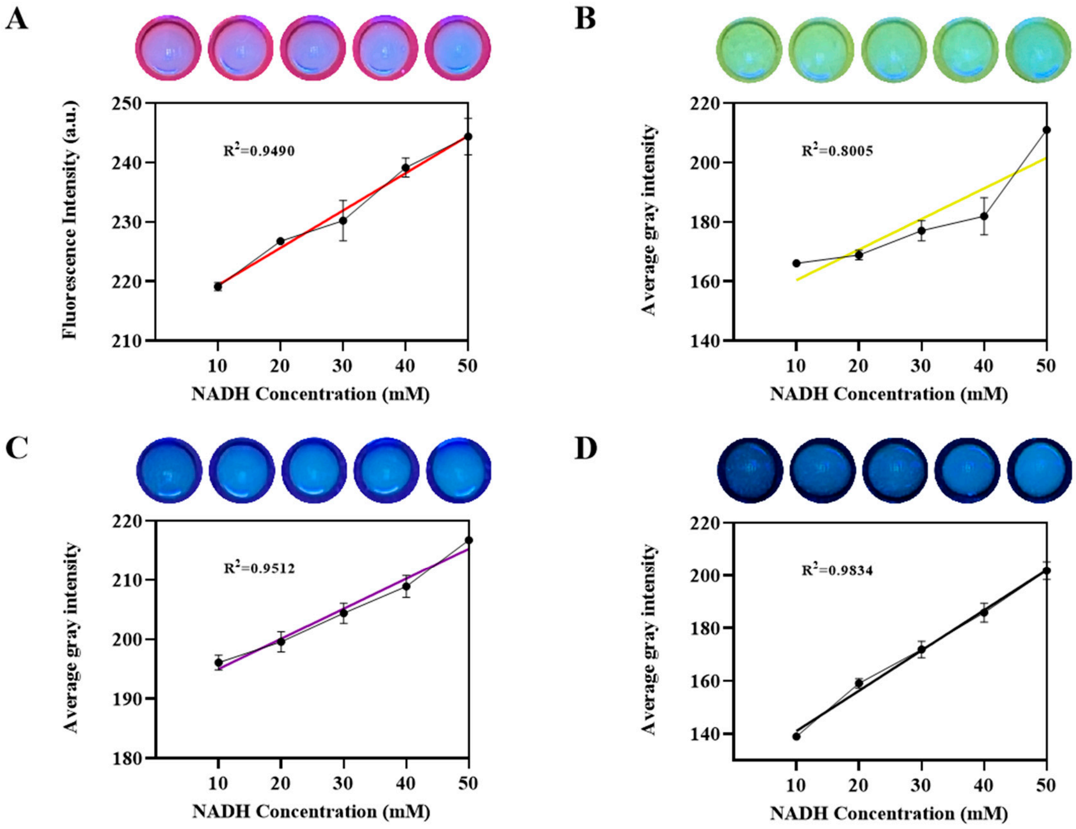

Figure S3. Color optimization of paper chip. The linear relationship and correlation coefficient between the NADH concentration and the corresponding gray value of fluorescence signal in the experimental group using red paper chip (**A**), yellow paper chip (**B**), purple paper chip (**C**) and black paper chip (**D**). All the evaluations were carried out in triplicate (n=3). ∗ represents a significant difference among the groups with $p < 0.05$.

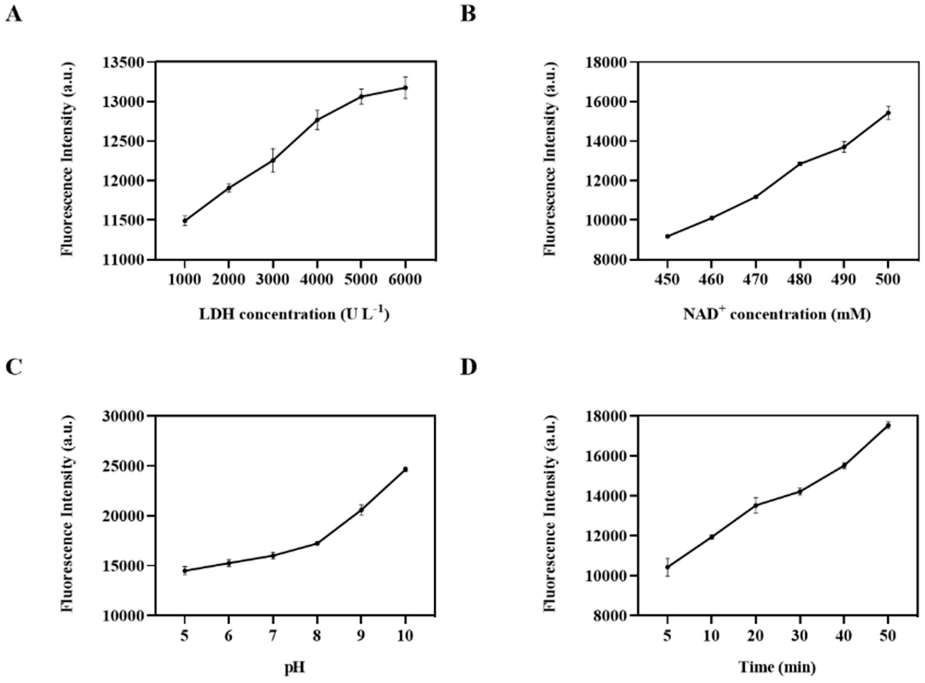

Figure S4. Conditions and fluorescence value curves of each experimental group in the microplate reader. (**A**) Optimization of LDH concentration. (**B**) Optimization of LDH concentration. NAD+ concentration. (**C**) Optimization of buffer pH value. (**D**) Optimization of reaction time. Error bars: standard deviation from different experiments (n = 3).

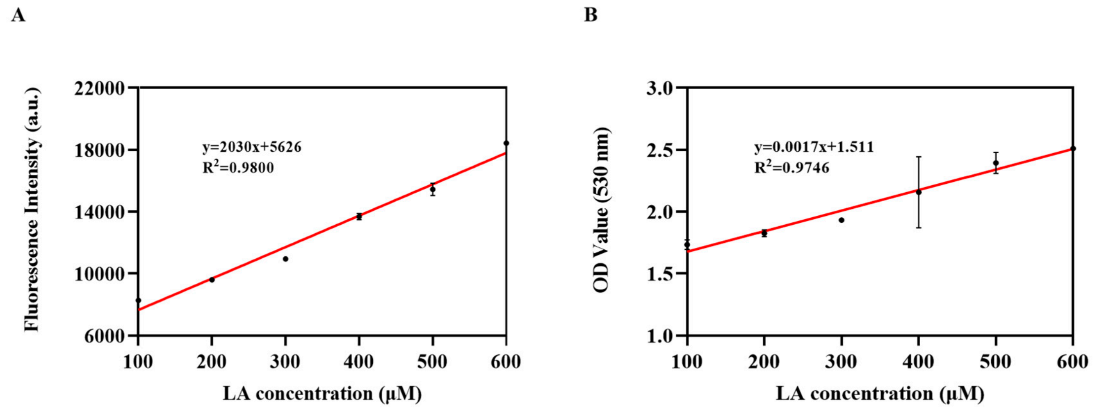

Figure S5. Calibration of the standard curve of fluorescence and lactate concentration using a microplate reader. Error bars: standard deviation from different experiments (n = 3). (**B**) Calibration of the standard curve of absorbance and lactate concentration in microplate wells using a commercial kit. Error bars: standard deviation from different experiments (n = 3).

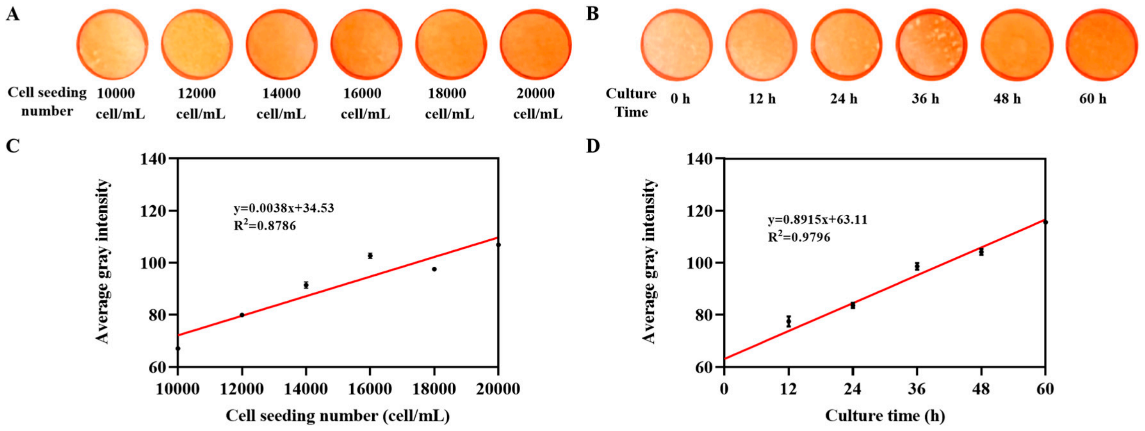

Figure S6. Feasibility verification of cell culture on paper-based platform (**A**) Calibration curve of different cell seeding densities and average gray intensity. (**B**) Calibration curve of cell culture days and average gray intensity. The average gray intensity was measured by the CCK-8 assay, and the image information was acquired by a smartphone. The results show that cell viability increases with increasing cell density. Considering the adhesion area of cells on paper, a seeding density of 16,000 cells/mL was chosen. As the culture time increased, the cell density also increased. To ensure sufficient cell density, cells were cultured for 24 h after seeding 16,000 cells for subsequent experiments. Additionally, it can be observed that our paper-based platform can be stored for at least 5 days for experimentation.All the evaluations were carried out in triplicate (n = 3).

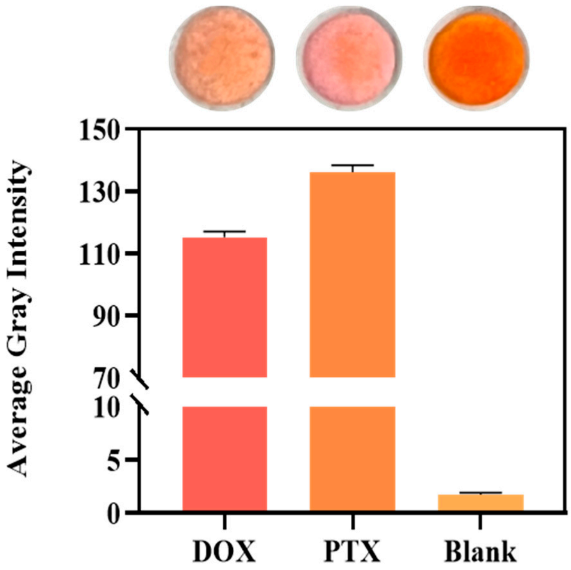

Figure S7. The cytotoxic effect of MCF-7 cells under the treatment of doxorubicin and paclitaxel： Cell viability detection after treatment with different drugs on a paper-based platform (images from left to right, doxorubicin, paclitaxel, concentration at 10μg mL^{-1}) ; Histogram showing grayscale intensity, collected by Image J from the images. All evaluations were repeated three times(n = 3).
